# Temperate Prophages Increase Bacterial Adhesin Expression and Virulence in an Experimental Model of Endocarditis Due to *Staphylococcus aureus* From the CC398 Lineage

**DOI:** 10.3389/fmicb.2019.00742

**Published:** 2019-04-24

**Authors:** Floriane Laumay, Anna-Rita Corvaglia, Seydina M. Diene, Myriam Girard, Frank Oechslin, Nathalie van der Mee-Marquet, José Manuel Entenza, Patrice François

**Affiliations:** ^1^Genomic Research Laboratory, Service of Infectious Diseases, Medical University Center, Geneva University Hospitals, Geneva, Switzerland; ^2^Unité de Recherche sur les Maladies Infectieuses et Tropicales Émergentes, Faculté de Médecine et de Pharmacie, Aix-Marseille University, Marseille, France; ^3^Department of Fundamental Microbiology, University of Lausanne, Lausanne, Switzerland; ^4^UMR 1282 Infectiologie Santé Publique, Université de Tours, Tours, France

**Keywords:** *Staphylococcus aureus*, bacteriophages (phages), virulence, infection, adhesion, internalization, endocarditis (infectious), lysogeny

## Abstract

Until 2007, *Staphylococcus aureus* from clonal complex 398 (CC398) was exclusively associated with livestock species and companion animals. Recently, several studies described the emergence of *S. aureus* CC398 as etiologies of severe infections in humans living in an animal-free environment. Recent sequencing efforts showed that the mobile genetic elements found in CC398 isolates were specific for each population and enabled differentiation of strains responsible for asymptomatic colonization from strains involved in bloodstream infections. We mobilized prophages from a human CC398 isolate and introduced them into two naïve ancestral isolates devoid of prophages that exclusively colonize animals. These lysogenized ancestral CC398 isolates acquired features related to virulence, such as an increased capacity to adhere to human extracellular matrix proteins and the ability to invade and survive within non-phagocytic cells. Pathogenicity of several clinical isolates from the CC398 lineage as well as ancestral and *in vitro* lysogenized ancestral counterparts was assessed in a model of infectious endocarditis in rats. Natural and artificial lysogens were not only more invasive than their prophage-free parent but also showed an increased capacity to multiply within aortic vegetations. This study identified prophages as mediators of bacterial virulence in a model of infectious endocarditis, probably through promotion of interaction with extracellular matrix components. Further studies are needed to identify mechanisms leading to promotion of intrinsic virulence.

## Introduction

Challenges related to *Staphylococcus aureus* infections in the human and veterinary clinics mobilized important human and technical resources. *S. aureus* can colonize 20–30% of the general population asymptomatically but is also capable of causing a wide spectrum of diseases ranging from benign infections, to particularly severe diseases such as endocarditis, necrotizing pneumonia, osteomyelitis, or septicemia (Lowy, 1998; Lew and Waldvogel, 2004). In milk-producing ruminants, mastitis caused by *S. aureus* is the major cause of economic loss worldwide and a major concern in milk transformation (Halasa et al., 2009).

The success of *S. aureus* as a pathogen is explained by its exceptional versatility and also by the plethora of virulence factors it is able to express (Lowy, 1998). Bacterial adhesins, notably, are proteins anchored within bacterial peptidoglycan and exposed to the extracellular medium, allowing interaction with host extracellular matrix proteins (Foster and Hook, 1998). The role of some major staphylococcal adhesins has been described *in vitro* and in experimental models of infections (McDevitt et al., 1994; Patti et al., 1994; Entenza et al., 2000; Elasri et al., 2002). In addition to adhesion, immune escape and the ability to form biofilms are important characteristics of *S. aureus* pathogenesis and persistence of colonization and infection (Costerton et al., 1999).

Most of the bacterial factors involved in infection severity are mobile genetic elements (MGEs) that belong to the accessory genome. Fitzgerald and colleagues were the first to describe that the genome of *S. aureus* is composed of approximately 80% of core-genes and 20% of accessory genes (Fitzgerald et al., 2001). MGEs include temperate bacteriophages stably integrated within the bacterial genome. Indeed, sequencing efforts showed that a large proportion of clinical isolates of *S. aureus* have one to several prophages within their genome. By supplying additional factors of virulence like the Panton-Valentine leukocidin, numerous enterotoxins, or by facilitating the exchange of genetic material between different strains, bacteriophages, and more generally MGEs, are known to carry potent toxins (Kraushaar et al., 2017) that contribute to *S. aureus* pathogenicity and to cellular tropism (Bae et al., 2006; Lindsay, 2010). In CC5 isolates, activation of the *sigB* regulon appears to be mediated by lysogenic phages (Fernandez et al., 2018). Ideally the identification of ancestral isolates devoid of mobile elements would be of particular interest for assessing virulence conferred by MGEs in an experimental model of infection.

Originally detected exclusively in pig farmers in a limited number of European countries (Verkade et al., 2012), *S. aureus* from the CC398 lineage is now a worldwide problem associated with livestock (Van Belkum et al., 2008; van der Mee-Marquet et al., 2014). An active surveillance program of bloodstream infections due to *S. aureus* was initiated in 2000 in our area. From this period, a constant increase in the prevalence of CC398 in patients living in animal-free environments was observed; the percent of *S. aureus* isolates responsible for severe infections went from 0% in 2007 to 15% in 2016 (Diene et al., 2017). This surveillance revealed a significant increase in strain virulence and the ability to spread from animal to human. Indeed, ancestral CC398 isolates were only able to colonize farm animals and evolved toward virulent strains able to infect humans without animal contact (referred to as the “emergent clade”) (Diene et al., 2017). Between these two distinct populations, some intermediate isolates were identified that are able to infect animals or to colonize humans. Important efforts that used whole genome sequencing were deployed on these different populations of strains. Sequencing the genomes of representative isolates showed that each population had its own specific prophage content (van der Mee-Marquet et al., 2014; Diene et al., 2017). Whereas the ancestral isolates associated with animals lacked any prophage element, emerging isolates are characterized by the presence of variants of the famous φ3 prophage that encodes two immune-modulating proteins known to alter or prevent chemotaxis, phagocytosis and killing of *S. aureus* by human neutrophils *in vitro*. These human-adapted isolates also harbor a φMR11-like-prophage inserted at the *smpB* locus. This bacteriophage contains a single gene encoding a putative superantigen similar to enterotoxin B but no characterized toxin showing potent effects on human cells. Using informatics tools, the nucleotide sequences of these prophages revealed that 50% of putative ORFs were annotated as a hypothetical protein with unknown function. We also noticed the presence of ORFs involved in phage processing and multiplication as well as phage repressor genes. In addition, we identified five single- or double-stranded DNA-binding proteins with unknown function.

The molecular mechanisms that evolved within the CC398 lineage to enable the carriage strains to become virulent are not known but are a particular subject of interest. To identify these molecular mechanisms, we mobilized prophages from a human CC398 clinical isolate and introduced the resultant particles into two naïve ancestral isolates devoid of prophages and non-human pathogen. Subjecting these “artificial lysogens” to several phenotypic tests, we were able to assess the contribution of prophages to bacterial virulence in different experimental models.

## Materials and Methods

### Control Strains and Clinical Isolates

All strains used in this study and their properties are described in Table 1. Most CC398 isolates were identified in patients suffering from bloodstream infection and belonged to the emergent clade. Bacteria were grown in Mueller-Hinton (MH) media at 37°C, unless stated otherwise.

**Table 1:** Strains and plasmids used in this study.

Strains		Source or references
***S. aureus***	**Characteristics**	
8325-4	NCTC 8325 cured of prophages and plasmids	Novick, 1967
DU5925	8325-4 *clfA* and *fnbp* deficient	Greene et al., 1995
8325-4 pCF4	8325-4 containing multicopy *clfA* plasmid	McDevitt et al., 1994
8325-4 pSKBIB	8325-4 containing multicopy *fnbp* plasmid	Greene et al., 1995
S123, S124	ST398 prophage-free ancestral isolates	van der Mee-Marquet et al., 2013a,b
S1	ST398 from human origin, livestock associated containing phages 2Pro and 3Pro (intermediate isolate)	van der Mee-Marquet et al., 2013b
S100	ST398 from human origin, non-livestock associated containing phages 4Pro and 5Pro (emergent clade)	Corvaglia et al., 2013
S123Sa2	S123 containing phages from S1	van der Mee-Marquet et al., 2013b
S124Sa2	S124 containing phages from S1	PRJEB31493
S92	ST398 from human origin, non-livestock associated containing 2Pro and other (emergent clade)	Diene et al., 2017
S13-192	ST398 from human origin, non-livestock associated containing 2Pro and other (emergent clade)	Diene et al., 2017
Cowan I	NCTC8530, septic arthritis isolate	ATCC 1298 (Sinha et al., 1999)
		
***S. epidermidis***		
*S. epidermidis* KH11		Peters et al., 1982

The human isolates were obtained from clinical samples during annual surveillance studies that were run in accordance with the French Healthcare recommendations for the prevention of infection. Ethical approval of the monitoring programs was obtained from the appropriate national committee: the “Réseau Alerte Investigation Surveillance des Infections Nosocomiales” (RAISIN). The methods and study design have been reported previously (van der Mee-Marquet et al., 2014). The study was managed jointly with the directors of the hospitals, the doctors responsible for patient care and the regional infection-control team.

### Genome Assembly

High-throughput sequencing was performed on purified genomic DNA obtained on DNeasy columns (Qiagen) and sequenced on an Illumina HiSeq 2500 (Illumina, San Diego, CA, United States) with 100-base paired-end reads and barcodes within the Nextera XT kit (Illumina), used in accordance with the manufacturer’s instructions. *De novo* genome assembly and annotation were performed as previously described (Diene et al., 2017).

### Phage Mobilization and Lysogenization Experiments

**FIGURE 1:**
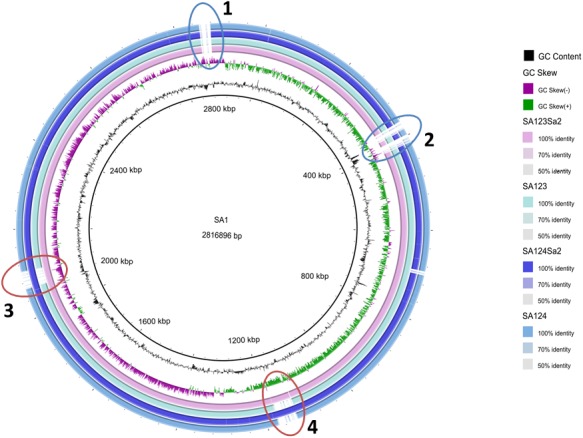
Circular representation of CC398 *Staphylococcus aureus* genomes. The figure was constructed using BRIG (http://brig.sourceforge.net/). The genome of strain S1 was used as reference and is the most inner circle. The GC content and skew for the S1 genome are shown in the second and third circles. The four outmost circles show the genome of S123Sa2 containing the two prophages and its parent S123, then strain S124Sa2 containing prophages and its parental isolate S124. All genomes were *de novo* assembled from data obtained from 100-base paired-end reads by using an Illumina HiSeq 2500 sequencer. Some regions present in S1 were absent or impossible to assemble in the four other isolates (1 and 2). Note that the prophages are inserted in the same regions in the donor strains as in the two *in vitro* lysogenized isolates (3 and 4). Note also that S123 and S123Sa2, and S124 and S124Sa2, differed only by the presence of the two prophages and did not differ at the single nucleotide level. A homology search identified ϕSLT and ϕ2956PVL similarities for phage 2Pro with a large central moiety homologous to ϕSa2wa, whereas phage 3Pro appeared to be partly homologous to ϕeta3 in its central section and similar to ϕX2 and ϕ80 for the extremities.

Strain S123Sa2 was obtained by introducing phages from S1 into S123. Strain S123 has been fully sequenced (van der Mee-Marquet et al., 2013b) and corresponds to the prophage-free ancestral sequence type 398 (ST398) isolate that is only able to colonize farm animals. Its genome is devoid of any prophage DNA. Phages from clinical isolate S1 were mobilized by adding mitomycin C to the growth medium as previously reported (de Gialluly et al., 2003). The two prophages showed partial homologies with viral particles identified in clinical isolates or in lab strains (see Figure 1). Supernatants from theses cultures were then filtered to remove cells and used to expose S123 to temperate phages. After incubation for 18 h at 30°C, suspensions were plated on fresh agar plates, incubated at 37°C overnight and screened for their prophage content by PCR using S1-phage specific primers. This led to the isolation and identification of prophage-containing strain S123Sa2, as previously described (van der Mee-Marquet et al., 2013a). Strain S124Sa2 was obtained following the same procedure performed on S124, an isolate from animals used as a recipient of prophages mobilized from strain S1. Similarly to S123, the genome of strain S124 is prophage-free as determined by PCR and sequencing (van der Mee-Marquet et al., 2013a).

### Bacterial Adhesion to Human Extracellular Matrix Proteins

Bacterial adhesion to solid phase-adsorbed human fibronectin, fibrinogen, collagen, serum or pooled plasma components was assessed in a 96-well plate format. Flat-bottom microtiter plates were coated for 1 h at 37°C with 50 μl per well of human fibronectin or fibrinogen or collagen at a concentration ranging from 0 to 40 μg/ml in PBS; human serum and pooled human plasma were used after diluting them 60-fold. The plates were washed three times with PBS and incubated at 37°C for 1 h after adding 250 μl of 2% HSA. The plates were rinsed three times with PBS prior being inoculated with a bacterial suspension prepared as follows. Bacterial isolates were inoculated into MH supplemented or not with antibiotic and incubated overnight at 37°C under constant agitation. Overnight cultures were 100-fold diluted in the same medium and incubated at 37°C for 1 h 30 or 3 h. Bacteria were washed with PBS and adjusted to an OD_600__nm_ = 0.9. 50 μl of the bacterial suspension was added to each well and after an incubation of 90 min at 37°C, the wells were rinsed three times with PBS. Adherent bacteria were fixed for 30 min at 60°C, stained with 95 μl of 0.5% crystal violet for 15 min and air-dried. Crystal violet staining was solubilized into 100 μl of DMSO and the absorbance measured at OD_595__nm_. In all experiments, negative and positive control strains were treated simultaneously as calibrators.

### Cellular Internalization in Human Embryonic Kidney Cells

HEK 293 cells were obtained from ATCC (CRL-1573), maintained in DMEM/Nut mix F-12 (Gibco), supplemented with 10% FCS, 50 IU/ml penicillin, and 50 μg/ml streptomycin. Cells were maintained in humidified air enriched with 5% CO_2_ at 37°C. Adherent 293 cells were plated in poly-ornithin-coated 24-well plates (diameter 1.7 cm) at around 2 × 10^5^ cells/well. Cells were washed with DMEM without FCS and antimicrobials. A volume of 0.5 ml of calibrated bacterial suspension in DMEM with 1% HSA and 10 mM HEPES was incubated with HEK cells for 3 h at 37°C, yielding a multiplicity of infection of 15:1. Cells were washed twice with PBS and 0.6 ml of lysostaphin (20 μg/ml) was added before incubation at 37°C for 20 min. After washing, HEK cells were lysed in 0.5 ml of H_2_O containing 0.2% Triton X-100 for 1–2 min before adding 0.5 ml of 0.9% NaCl. Cellular lysates were then serially diluted and plated on MH agar plates. CFUs were quantified manually or using a laser counter (Countermat, IUL Instrument, Germany) when it was possible. In all experiments, negative and positive control strains were treated simultaneously as calibrators (Sinha et al., 1999).

### RNA Isolation and qRT-PCR

RNA qRT-PCR was performed as described previously (Fischer et al., 2011) from cell suspensions grown at 37°C for 3 h in MH from a 1:500 dilution of overnight culture (Garzoni et al., 2007). DNA was removed by twofold DNase I digestion as described previously (Garzoni et al., 2007; Fischer et al., 2011). A batch of 200 ng of total RNA for each strain was subjected to reverse-transcription using SuperScript II (Invitrogen, Basel, Switzerland). A volume of 5 μl of a 1:10-fold dilution was used for quantitative PCRs (qPCRs). Gene-specific primers and 6-carboxyfluorescein (FAM)-coupled probes (Li et al., 2005; Fischer et al., 2011) for *hu* and tested genes [except *trap* and RNAIII that used a SybrGreen assay (Li et al., 2005; Fischer et al., 2011)] were mixed with qPCR reagents (Thermo Fisher Scientific/Agilent Technologies). Amplifications were performed in a Bio-Rad CFX96 and normalized using intensity levels recorded for the *hu* gene as described by our group (Garzoni et al., 2007). Replicates included RNAs that were extracted two independent times, as well as two independent qPCR reactions.

### Rat Experimental Endocarditis Model

The rat endocarditis model experiments were carried out in strict accordance with the recommendations of the Swiss Federal Act on Animal Protection. The protocol was approved by the Committee on the Ethics of Animal Experiments of the Consumer and Veterinary Affairs Department of the State of Vaud (Permit No. 879.9). Aortic vegetations were produced in female Wistar rats as described previously by using catheter (Moreillon et al., 1995). Groups of rats (8–11 rats per group) were inoculated intravenously (i.v.) with 10^4^ CFUs with exponential phase cultures. This inoculum size was used because is the minimal inoculum producing/inducing endocarditis in 50% of the animals challenged with the S123 prophage-free ancestral isolate strain, and thus permitted detection of infection by more-virulent strains. After 24 h of inoculation, animals were sacrificed and quantitative vegetation culture was performed on blood agar plates. Bacterial titers, expressed as the mean of log_10_ CFUs/g of tissue, were determined on agar medium following incubation for 48 h at 37°C.

### Statistical Analysis

The adhesion of the different isolates to human extracellular matrix proteins and fibrinogen coated surfaces as well as the internalization in HEK cells was compared by using a Mann–Whitney *U*-test, whereas Fisher’s exact test was used to compare the levels of infected vegetations. Mean bacterial titers in vegetations were compared using the unpaired Student’s *t*-test. All statistical analyses were performed with GraphPad Prism software (version 6.0 for Windows; GraphPad Software, La Jolla, CA, United States). Differences were considered significant at *P*-values of < 0.05, by use of two-sided significance levels.

## Results

### Bacterial Genome Organization and Phage Content

All strains used in this study (Table 1) belong to CC398 as determined using multi-locus sequence-typing. Strains S123 and S124 are ancestral isolates that asymptomatically colonize farm animals and are devoid of prophages, as shown by whole genome sequencing and phage-identifying PCR (van der Mee-Marquet et al., 2013a,b). S1 was identified from a pig farmer (i.e., human colonizing, intermediate isolate) and its genome sequence revealed the presence of two prophages (Diene et al., 2017), namely 2Pro and 3Pro. The genome sequence of S123Sa2 -a strain lysogenized with prophages mobilized from S1- showed that the two prophages from S1 are integrated at the same locations they occupied in the S1 parental strain; this was an intergenic region between *tmRNA* and a hypothetical protein for phage 3Pro and in *ecsB* (a multidrug transporter) for phage 2Pro. *tmRNA* was not disrupted and no difference in strain pigmentation was observed. Phage 2Pro was 45.57-kb and 33.34% GC whereas phage 3Pro was 41.39-kb and 35.6% GC. Note that we failed to obtain a strain showing single phage insertion, a phenomenon already observed in other bacterial species (Hassan et al., 2010). Circular representation of a whole genome alignment (Figure 1) showed that despite minor differences between the donor strain S1 and receiver strains S123 and S124, we were able to visualize that the insertion of each phage was unique and at the same location in the donor strain and the two *in vitro* lysogenized isolates S123 and S124 (Figure 1). No difference was recorded by precisely comparing pairs of strains S123/S123Sa2 and S124/S124Sa2 except the presence of the two prophages. None of the genes flanking the insertion regions are known to be involved in major regulatory processes affecting *S. aureus* pathogenesis, except the gene encoding TRAP in S123Sa2, which is known to interact with the *agr* locus. However, *trap* and *rnaIII* (*agr*) expression was similar in S123 and its counterpart S123Sa2 (Table 2). In addition, no sequence differences were found in regulators of virulence determinants (*agr*, *rnaIII*, *sarA*, and all *sar* homologs, *sigB*, *rsbUVW*, *trap*, *svrA*, *saeRS*) or metabolic regulators (*ccpA*, *codY*, *glnR*, *nreC*, *rex*, *srrAB*, *mgrA*, *perR*, *fur*, *ideR*, *zur*) when S123 and S123Sa2, as well as S124 and S124Sa2, were compared. None of these prophages had any impact on growth rate or on the general antimicrobial resistance profile (data not shown).

**Table 2:** Ratios of gene expression between the lysogenic strains and their parents.

Genes	Mean fold-change ± SEM	
	**S123Sa2/S123**	**S124Sa2/S124**
*clfA*	2.1 ± 0.2	1.2 ± 0.2
*fnbpA*	6.7 ± 0.8	2.0 ± 0.3
*cna*	1.3 ± 0.1	2.1 ± 0.1
*trap*	1.2 ± 0.1	ND
*agr* (RNAIII)	0.7 ± 0.1	ND

*Data are expressed as the mean ratio value of fold change ± range obtained by comparing cycle of quantification (CQ) in S123Sa2 or S124Sa2 to CQ of the corresponding parent S123 or S124 (control strains). The level of transcription of the hu gene was used to normalize RNA amounts. The values represent averages of duplicate determinations from two independent experiments.*

### Interaction With Surfaces Coated With Extracellular Matrix Proteins

**FIGURE 2:**
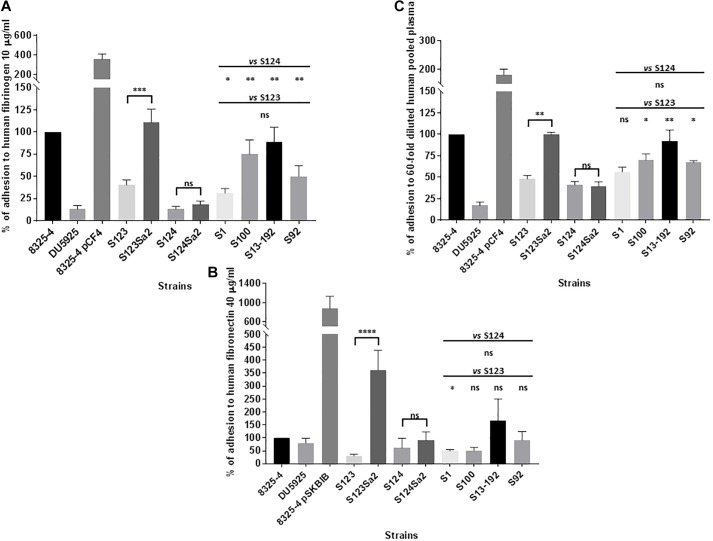
Adhesion to extracellular-coated surfaces. Adhesion of *Staphylococcus aureus* to human fibrinogen-coated **(A)** fibronectin-coated **(B)** and pooled plasma -coated **(C)** flat bottom 96-well plates after 3 h of growth in MH. Control strains included: strain 8325-4 with its clumping factor A and fibronectin binding proteins A and B (ClfA, Fnbp A and B), mutant DU5925, strain 8325 pCF4 harboring a multicopy plasmid encoding ClfA, and strain 8325-4 pSKBIB containing a multicopy plasmid encoding Fnbp B. The OD_595__nm_ was read as the mean value for a given isolate after subtraction of the OD_595__nm_ for bacteria that were incubated with PBS only, and after division by the OD_600__nm_ of the corresponding bacterial suspension. Normalized values were expressed as a percentage of the value for the positive control (8325-4) on the same plate. The results represent mean ± SEM of at least three independent experiments (eight replicates for fibrinogen, four for fibronectin, and three for pooled plasma). ^∗∗∗∗^*P* < 0.0001 by Mann–Whitney test; ^∗∗∗^*P* < 0.001; ^∗∗^*P* < 0.01; ^∗^*P* < 0.05.

Figure 2 shows the adhesion of control positive (8325-4), negative (DU5925), and hyper-adherent (8325-4 pCF4/8325-4 pSKBIB) isolates to surfaces coated with human fibrinogen or fibronectin. Lysogenized strains S123Sa2 and S124Sa2 showed an increase in adhesion to human fibrinogen compared to their prophage-free parents S123 and S124 (3- and 1.4-fold respectively; Figure 2A), whereas phage donor isolate S1 showed average adhesion capacity. A stronger difference was noted for adhesion to human fibronectin, where strains S123Sa2 and S124Sa2 showed an increase of 12- and 1.5-fold respectively, compared to that of their parental strains (Figure 2B). Regarding the phenotype of S124, its fibronectin-binding protein A sequence contained several alterations (Supplementary Figure S1) compared to the homologous sequence in S123 (containing a functional FnpbA protein), which may explain its relatively poor adhesion. In addition, we were not able to reliably assemble the *clfA* gene in S124 using reads from the Illumina run. However, we were able to assess transcript levels, as our primers/probes matched the gene in its 5′ region. Note also that natural lysogens (S100, S13-192, S92), when compared to “clonal” prophage-free isolates, showed a moderate ability to adhere to a fibronectin-coated surface (Figure 2B), and a higher capability to adhere to fibrinogen and to pooled plasma components (Figure 2A).

Adhesion to human serum components was minimal for all strains whereas pooled human plasma components -probably related to fibrinogen- promoted a twofold higher adhesion of S123Sa2 compared to its parent S123 (Figure 2C). No significant differences between lysogens and prophage-free strains were detected in adhesion to a surface coated with human type I collagen.

The same set of strains was tested for biofilm formation on a polyethylene surface. Similar levels of biofilm were recorded for all isolates, regardless of whether they were a parental strain, an *in vitro*-lysogenized strain, or a human adapted isolate (not shown). Prophage-free and lysogenic strains had similar antibiotic susceptibility profiles and generation times (36–38 min).

### Bacterial Internalization Into Non-phagocytic Cells

**FIGURE 3:**
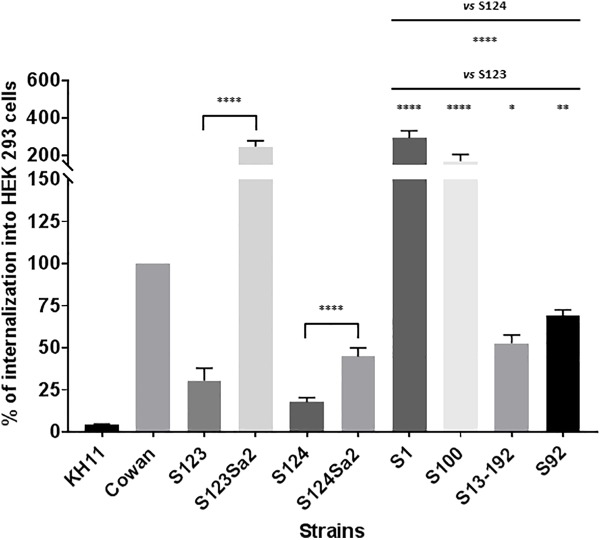
Internalization of *Staphylococci* in HEK cells. Internalization was measured by using a lysostaphin protection assay. HEK cells and bacteria were incubated for 3 h before the addition of lysostaphin. CFU counts following lysostaphin treatment were normalized with CFU counts from the initial inoculum. The results represent the mean ± SEM of three independent experiments and are expressed as a percentage of *S. aureus* Cowan I internalization. ^∗∗∗∗^*P* < 0.0001 by Mann–Whitney test; ^∗∗∗^*P* < 0.001; ^∗∗^*P* < 0.01; ^∗^*P* < 0.05.

Strain Cowan I was used as positive control and has been shown to invade endothelial or epithelial cells (Sinha et al., 1999). In contrast, the negative control KH11 showed only a marginal level of internalization. Similar to KH11, the ancestral CC398 strains S123 and S124 showed only limited internalization compared to lysogenized mutants S123Sa2 and S124Sa2 (8- and 2.5-fold lower respectively, see Figure 3). The levels of internalization obtained for the strain used as donor (S1) and representative clinical isolates of human origin also indicated these strains were able to invade HEK cells.

### Level of Transcription of Global Regulators and Adhesin Genes

Transcription levels of bacterial adhesins and global regulators were assessed by using quantitative RT-qPCR on purified RNAs obtained from strains grown in conditions identical to those used for the adhesion assays. Expression of *trap* in strain S123 and S123Sa2 was similar and that of RNAIII was slightly decreased (not significant). In contrast, the expression level of *fnbpA* -encoding the main adhesin responsible for adhesion to fibronectin- differed between strains S123 and S123Sa2 with higher transcription levels in the *in vitro*-lysogenized isolate. Expression of the *clfA* gene, which is responsible for adhesion to fibrinogen, was also higher in S123Sa2 compared to S123 (Table 2). Moderate increases were also observed for *fnbpA* and *cna* in S124Sa2 compared to S124.

### Assessment of Virulence in an Experimental Model of Infectious Endocarditis in Rats

**FIGURE 4:**
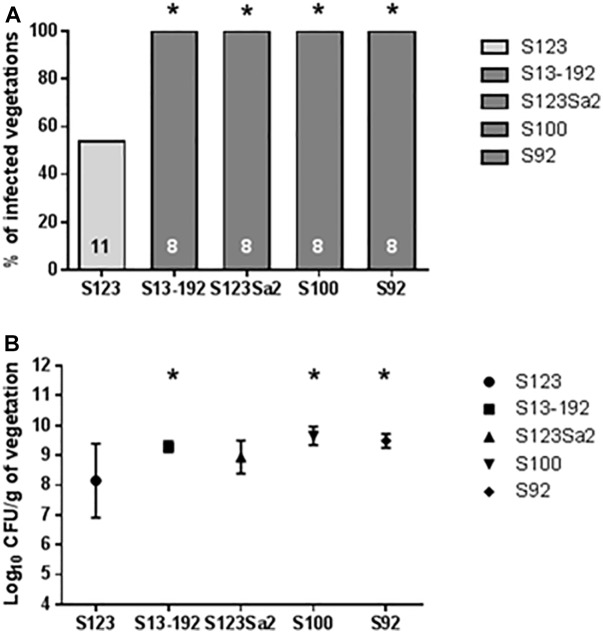
Results of experimental endocarditis. Incidence of endocarditis **(A)** and bacterial densities in infected vegetations **(B)** in rats 24 h after inoculation with 10^4^ CFUs of the tested *S. aureus*. The numbers of animals in each group are indicated at the bottom of the columns. Bacterial densities in **(B)** are expressed as mean ± SD. ^∗^ Indicates values that were significantly different (*P* < 0.05) from values for strain S123 by using either the Fisher’s exact test (infected vegetations) or the Student’s *t*-test (bacterial densities).

At an infecting titer of 10^4^ CFUs, the prophage-free ancestral strain S123 produced vegetation infection in only 6/11 (around 50%) cases 24 h after inoculation. In contrast, strains containing phages 2Pro (strains S1, S92, and S13-92) and 3Pro (strain S1) or 4Pro and 5Pro (strain S100) yielded an infection rate of 100% (eight infected animals; *P* < 0.05). Strain S123 lysogenized with phages from strain S1 (i.e., S123Sa2) also showed a statistically significant increase in infection (*P* < 0.05) compared to its prophage-free parent S123 (Figure 4A).

An important parameter obtained from the experimental endocarditis model was also the number of CFUs recovered from the vegetations (Figure 4B). The lowest number of bacteria in vegetations was obtained for the prophage-free strain S123. In comparison, all other isolates containing prophages showed higher bacterial vegetation titers.

## Discussion

*Staphylococcus aureus* is responsible for a wide diversity of infections showing a large range of severity (Lowy, 1998). Some staphylococcal toxins have been shown to directly mediate specific infections such as TSST-1 in toxic shock syndrome or enterotoxins for food-poisoning infections. However, virulence factors involved in chronic infections are not clearly identified. The clinical observations involving *S. aureus* from the CC398 lineage are particularly interesting from evolutionary and epidemiological perspectives. Until recently, this lineage was exclusively found in animals or in humans in close contact with animals. This lineage evolved and is now responsible for severe infections in humans whether or not they have contact with animals. Our group and others have shown that this evolution from animal to human host and the increase in virulence are mediated by the acquisition of MGEs (Price et al., 2012; van der Mee-Marquet et al., 2014; Diene et al., 2017).

Following an annual survey of all bloodstream infections in a large population area covering >3 million inhabitants, we reported a significant increase in the prevalence of strains from the CC398 lineage (Diene et al., 2017). In the course of previous studies performed with veterinarians from Netherlands we identified two animal CC398 isolates devoid of MGEs. We used these two strains as “naïve receivers” of bacteriophages obtained from a human colonizing clinical isolate of the same lineage. Whole genome sequencing revealed that the two prophages have genome sizes around 45–50 kb with GC contents slightly higher than those of the bacterial genomes. Each phage genome had 50–55 open-reading frames that mostly encoded “protein with unknown function,” and also encoded several proteins with a putative DNA binding domain.

*Staphylococcus aureus* is frequently responsible for foreign body and catheter-related infections through the formation of biofilm or interaction with extracellular matrix proteins (Lowy, 1998). Notably, fibrinogen-binding and fibronectin-binding proteins have been shown to contribute to infective endocarditis and indwelling device infections (Hienz et al., 1996; Francois et al., 1998; Que et al., 2001, 2005).

Our study demonstrated that integration of prophages in naïve isolates resulted in increased transcription of *clfA* and *fnbA*, which resulted in increased levels of adhesion to plasma-, fibrinogen-, or fibronectin-coated surfaces. The changes in fibronectin-adhesion in S124 following prophage insertion were consistent across multiple measurements: a twofold increase in transcription, a 1.5-fold increase in adhesion and a 2.5-fold increase in internalization. Similarly, in the strain pair S123/S123Sa2 we recorded a 6.7-fold increase in transcription, a 12-fold increase in fibronectin adhesion and an eightfold increase in internalization. Importantly, alteration of transcription was not due to the insertion of a prophage near *trap* (or in the vicinity of any global regulator), whose expression was similar in both strains. This increase in the expression of adhesins, particularly fibronectin-binding proteins, could explain the significant increase in the ability to be internalized in non-phagocytic cells (Sinha et al., 1999), allowing the bacterium to reside in a protected environment not accessible to host defenses or to most antibiotics used in clinics. The adhesion phenotype also affected the severity of endocarditis in a rat model of infection (Que et al., 2001, 2005), an infection model where fibrinogen- and fibronectin-binding proteins contributed to the cardiac valve colonization and endothelial cell invasion by *S. aureus* (Que et al., 2005). Fibrinogen appears to be a critical factor for initial attachment, whereas fibronectin-binding factors mediate alteration in bacterial clearance by the host immune system (Que et al., 2005) and also play a role in the internalization by non-phagocytic cells (Sinha et al., 1999, 2000). This phenotypic alteration required functional adhesin genes and resulted in increased virulence features. Other lysogens (S100, S13-192, S92) were slightly more adherent to fibrinogen compared to their prophage-free parent and adhered only moderately to fibronectin. However, lysogenization increased each strain’s capability to invade human non-phagocytic cells, as well as its capacity to colonize aortic vegetations in our model of endocarditis. Note however that the impact of prophages on adhesin gene expression was observed even in the S124 strain containing mutations, since its counterpart S124Sa2 displayed a slight overexpression of these genes.

Precise analysis of all ORFs harbored by the prophages failed to detect any LPXTG-containing proteins, suggesting the probable absence of additional cell wall associated molecules able to mediate additional interactions with host matrix proteins. In addition, integration of the two prophages only led to the disruption of a multidrug transporter -without altering the susceptibility profile of the strain- and did not disrupt any potential regulators. One prophage was integrated near *clfA* but distant from *fnbp* genes. Our hypothesis for future research is that the presence of one or more uncharacterized transcription regulators carried by the prophages results in altered transcription of important bacterial adhesins. It would represent the first precise molecular mechanism involving a prophage gene that regulates a bacterial virulence gene.

Emerging human pathogens can originate from microbes that normally infect other animals. As an illustration, a human-to-animal jump has been observed after acquisition of MGEs from avian specific lineages, in addition to inactivation of specific proteins required for human infection leading to the occurrence of a poultry-specific ST5 clade (Murray et al., 2017). Conversely, the major bovine *S. aureus* clonal complex CC97, predominantly identified in ruminants in the past, has recently caused human infections. The emergence of a new human-infecting CC97 clone may be related to the acquisition of a MGE that encodes factors for adaptation to the human host (Spoor et al., 2013). In addition, the CC9 lineage that usually infects only pigs has been increasingly implicated in bloodstream infections in humans not living near livestock (Lamamy et al., 2013).

Prophages have critical roles in bacterial diversity and evolution. They modulate bacterial virulence and contribute to microbial evolution by carrying genes encoding for novel information provided to their host. Our study suggests that prophages have a direct impact on bacterial virulence and bacterial pathogenicity. The identification of mechanisms triggering this phenomenon should be studied at the molecular level.

## Conclusion

Introduction of bacteriophages from human-adapted *S. aureus* to a naïve animal colonizer resulted in virulent strains with altered transcription levels of important bacterial adhesins. Furthermore, the presence of bacteriophages conferred increased virulence in a model of infectious endocarditis for all lysogens tested.

## Author Contributions

FL, A-RC, and MG performed the adhesion and internalization experiments. A-RC and NM-M performed the transduction experiments. SD performed genome assembly and comparisons. FO and JE performed endocarditis experiments. NM-M and PF designed the study. FL, NM-M, and PF were also involved in manuscript writing.

## Conflict of Interest Statement

The authors declare that the research was conducted in the absence of any commercial or financial relationships that could be construed as a potential conflict of interest.
