# Supplementary material for: Temperate Prophages Increase Bacterial Adhesin Expression and Virulence in an Experimental Model of Endocarditis Due to Staphylococcus aureus From the CC398 Lineage
Source: Front Microbiol. 2019 Apr 24;10:742. doi: 10.3389/fmicb.2019.00742 (PMC6492496; doi:10.3389/fmicb.2019.00742)
Supplement: Supplementary file 1 [file Table_1.DOCX]

**Supplementary Figure S1**


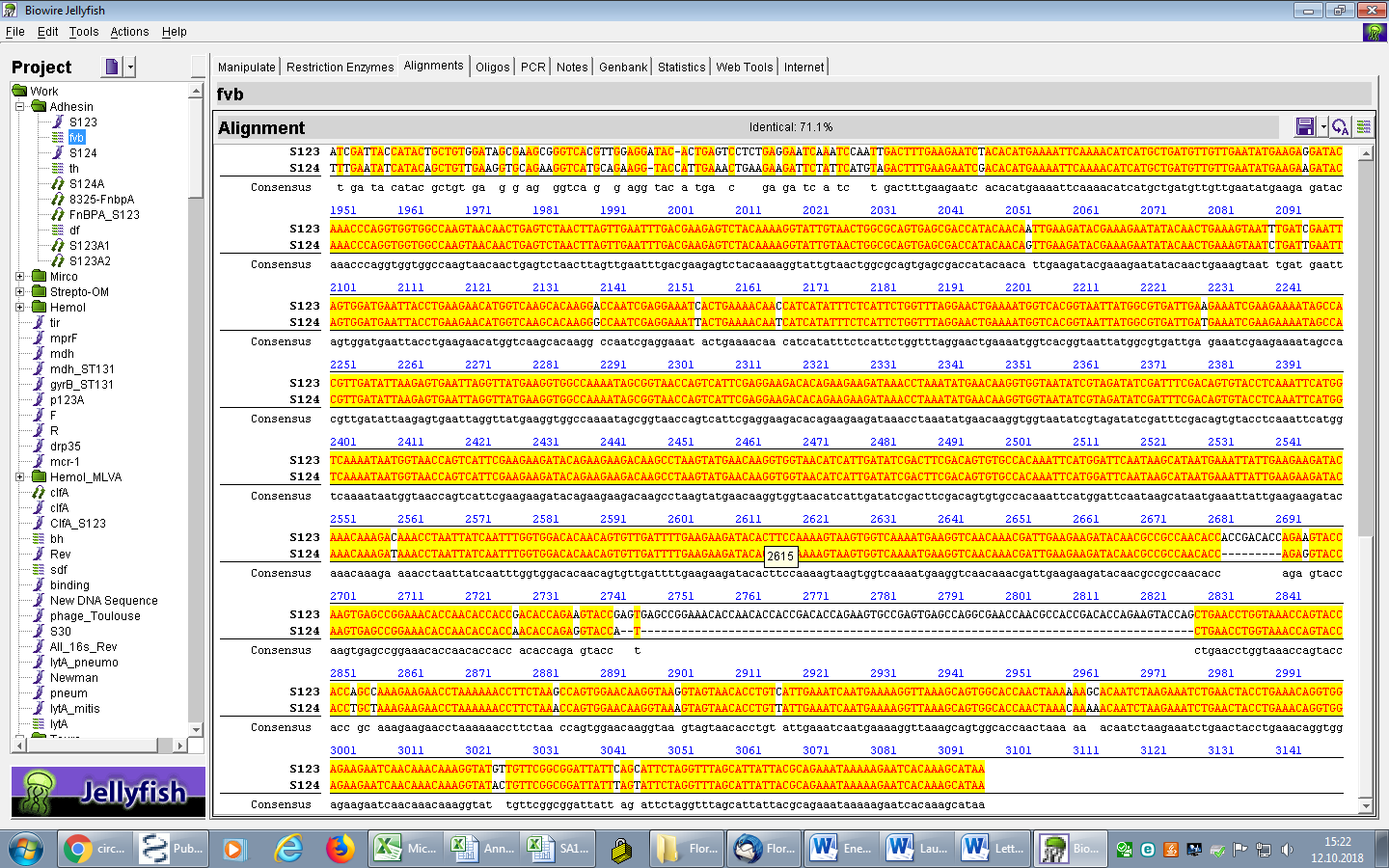


Blast comparison of predicted fibronectin-binding protein A gene from S123 and S124 showing alteration of the sequence in S124, probably leading to defective function of the protein.
